# Unusual Effects of Hyoscine

**Published:** 1895-04-06

**Authors:** 


### UNUSUAL EFFECTS OF HYOSCINE.

Dr. Fiske (N.Y. Med. Record, July 1893), contributes the notes of a case in which he treated insomnia, due partly to alcoholic indulgence and partly to business worries, with hypodermic injections of hyoscine hydrobromate. The drug, as is well known, is now rather extensively used as a hypnotic and sedative in delirium tremens, mental excitement, insomnia, &c. The patient was a healthy man, forty years of age, and when first seen was very restless, sleepless, and excited. At eleven p.m. he got $\frac{1}{100}$ grain hyoscine hydrobromate without effect, and the dose was repeated in three-quarters of an hour. He dosed for about half an hour, and then relapsed into his former condition of extreme restlessness, which continued until early next morning, when he was again seen by Dr. Fiske. At eight a.m. $\frac{1}{50}$ grain more was given. Shortly after he experienced numbness in his extremities, his gait was affected, his hands became purplish, his voice was husky, and his legs so weak that he fell on attempting to walk. The pulse was 90 per minute. In two and a half hours he had recovered, but had never felt any inclination to sleep. Morphine, $\frac{1}{2}$ grain, hypodermically, obtained him about thirty minutes sleep. At five p.m. $\frac{1}{50}$ grain hyoscine hydrobromate was again given, when he had similar symptoms to the above, and his pulse rose to 112 per minute; he recovered in about three hours, but never experienced any desire to sleep. He had hallucinations of faces being around him, and engaged in conversation with imaginary persons. Large doses of chloral hydrate and bromides produced no satisfactory sleep. Most probably the specimen of hyoscine consisted chiefly of hyoscyamine, several alkaloids are obtained from the hyoscyamus niger and it has been repeatedly pointed out that the hyoscine of commerce is frequently impure.

